# Can multitrophic interactions and ocean warming influence large‐scale kelp recovery?

**DOI:** 10.1002/ece3.4963

**Published:** 2019-02-14

**Authors:** Hartvig Christie, Hege Gundersen, Eli Rinde, Karen Filbee‐Dexter, Kjell Magnus Norderhaug, Torstein Pedersen, Trine Bekkby, Janne K. Gitmark, Camilla W. Fagerli

**Affiliations:** ^1^ Norwegian Institute for Water Research Oslo Norway; ^2^ Department of Biology University of Oslo Oslo Norway; ^3^ Institute of Marine Research, Flødevigen His Norway; ^4^ Department of Arctic and Marine Biology Arctic University of Norway Tromsø Norway

**Keywords:** crabs, kelp, mesopredator release, ocean warming, predator control, regime shift, sea urchins

## Abstract

Ongoing changes along the northeastern Atlantic coastline provide an opportunity to explore the influence of climate change and multitrophic interactions on the recovery of kelp. Here, vast areas of sea urchin‐dominated barren grounds have shifted back to kelp forests, in parallel with changes in sea temperature and predator abundances. We have compiled data from studies covering more than 1,500‐km coastline in northern Norway. The dataset has been used to identify regional patterns in kelp recovery and sea urchin recruitment, and to relate these to abiotic and biotic factors, including structurally complex substrates functioning as refuge for sea urchins. The study area covers a latitudinal gradient of temperature and different levels of predator pressure from the edible crab (*Cancer pagurus*) and the red king crab (*Paralithodes camtschaticus*). The population development of these two sea urchin predators and a possible predator on crabs, the coastal cod (*Gadus morhua*), were analyzed. In the southernmost and warmest region, kelp forests recovery and sea urchin recruitment are mainly low, although sea urchins might also be locally abundant. Further north, sea urchin barrens still dominate, and juvenile sea urchin densities are high. In the northernmost and cold region, kelp forests are recovering, despite high recruitment and densities of sea urchins. Here, sea urchins were found only in refuge habitats, whereas kelp recovery occurred mainly on open bedrock. The ocean warming, the increase in the abundance of edible crab in the south, and the increase in invasive red king crab in the north may explain the observed changes in kelp recovery and sea urchin distribution. The expansion of both crab species coincided with a population decline in the top‐predator coastal cod. The role of key species (sea urchins, kelp, cod, and crabs) and processes involved in structuring the community are hypothesized in a conceptual model, and the knowledge behind the suggested links and interactions is explored.

## 1 INTRODUCTION

Transitions between kelp forests and sea urchin‐dominated barren grounds have been studied for decades (reviewed by Filbee‐Dexter & Scheibling, 2014; Lawrence, 1975). Both kelp forests and barrens have been described as stable states (Elner & Vadas, 1990; Marzloff et al., 2013), with several reinforcing feedback mechanisms, making a shift to the alternative state difficult (Filbee‐Dexter & Scheibling, 2014; Ling et al., 2015). The existence of different critical thresholds for the shifts back and forth (i.e., hysteresis, Scheffer, Carpenter, Foley, Folke, & Walker, 2001) has been suggested (Ling et al., 2015). Drivers of shifts from barrens back to kelp seem to vary among regions and include changes in predation pressure (Estes, Tinker, & Williams, 1998; Fagerli, Norderhaug, Christie, Pedersen, & Fredriksen, 2014), stochastic events such as diseases (Scheibling, Hennigar, & Balch, 1999), El Niño events (Vásquez, Vega, & Buschmann, 2006), and climatic extremes (Fagerli, Norderhaug, & Christie, 2013; Rinde et al., 2014).

**Figure 1:**
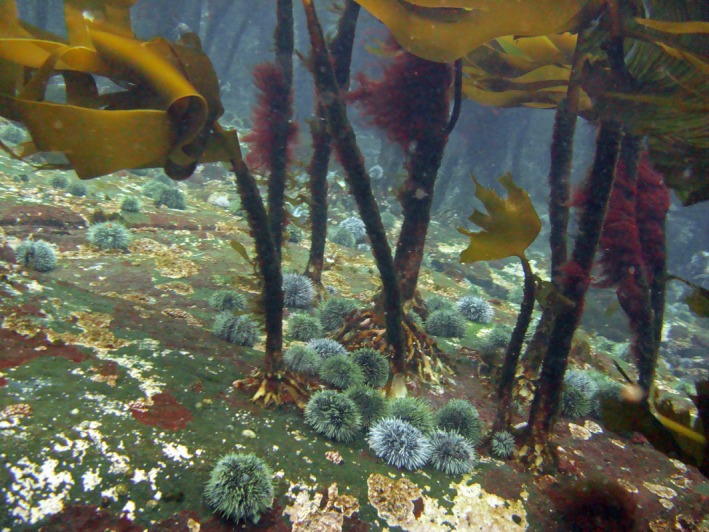
Photograph from Hammerfest in northern Norway, showing newly recovered *Laminaria hyperborea* where sea urchins *Strongylocentrotus droebachiensis* still are present (photograph: H. Christie)

In the northeastern Atlantic, studies on the dynamics of kelp forests and urchin barrens have largely focused on the extent of the areas affected by overgrazing, the large annual loss of kelp production (millions of tons), the loss of habitats for commercial fish and other species, and the resilience of the two states facing different stressors (Figure 1, Norderhaug & Christie, 2009). Currently, wide‐scale kelp recovery is occurring along distinct regions of the Norwegian coast (Norderhaug & Christie, 2009; Rinde et al., 2014), offering a rare opportunity to explore important scientific and management questions related to the processes and mechanisms involved in the return and persistence of recovered kelp forests.

For more than four decades, the green sea urchin *Strongylocentrotus droebachiensis* has persisted in high densities between 63°N and 71°N along the mid‐ and northern Norwegian coast and into Russian waters in the northeast. In the 1970s–1980s, sea urchin grazing reduced kelp abundance and biomass all along this coastline (Gudimov, Gudimova, & Pavlova, 2003; Norderhaug & Christie, 2009; Propp, 1977; Sivertsen, 1997, 2006), and hundreds of square kilometers of highly productive kelp forests (*Laminaria hyperborea* in exposed waters and *Saccharina latissima* in more sheltered areas) were replaced by sea urchin‐dominated barren grounds, resulting in a massive loss of habitat, diversity, and production at different trophic levels (Christie, Norderhaug, & Fredriksen, 2009; Leclerc, Riera, Leroux, Levenque, & Davoult, 2013; Norderhaug, Christie, Fossa, & Fredriksen, 2005; Pedersen, Nejrup, Fredriksen, Christie, & Norderhaug, 2012). The barrens were maintained for years through regular sea urchin recruitment (Fagerli et al., 2013) and by the sea urchins’ ability to grow and survive even with a low food supply (Russell, 1998; Russell, Ebert, & Petraitis, 1998). From the 1990s, however, sea urchin densities have decreased, resulting in the recovery of large areas of kelp forest in the southern parts of the coast (Norderhaug & Christie, 2009; Skadsheim, Christie, & Leinaas, 1995), and reports of local recovery of kelp forests in the north, near the Russian border (pers. comm. Norwegian fishers, see also Gudimov et al., 2003).

Studies (e.g., Steneck, Vavrinec, & Leland, 2004; Steneck, Leland, McNaught, & Vavrinec, 2013) suggest that important processes driving kelp forest recovery in the Atlantic include a combination of changing environmental conditions and multitrophic top‐down control on sea urchins. In this area, sea temperatures have increased at a rate between 0.03 and 0.04°C per year over the last 40–50 years (Fagerli et al., 2013), influencing kelp recovery in several ways. The green sea urchin is a cold‐water species (Siikavuopio, Christiansen, & Dale, 2006; Siikavuopio et al., 2012), and the observed sea urchin population decline in mid‐Norway has been associated with sea water temperature increases above a critical threshold (Fagerli et al., 2013; Rinde et al., 2014), which negatively impact sea urchin larval development (Stephens, 1972). This indicates that kelp recovery can be triggered by both a gradual increase in temperature above the critical threshold or by stochastic events exceeding the threshold. In contrast, the edible crab *Cancer pagurus* and the less studied *Carcinus maenas*, both important sea urchin predators (Fagerli et al., 2014), are expanding northward (indicated by catch rate data by Woll, Meeren, & Fossen, 2006). This is likely a result of warmer waters (Woll et al., 2006), an assumption that has been supported by Lindley and Batten (2002) and Lindley and Kirby (2010), who documented a northward expansion of decapod larvae as temperature increased. Along the northernmost Norwegian coast (70°N), temperatures are cold and sea urchin recruitment is high (Fagerli et al., 2013, 2015). It is therefore unlikely that temperature‐driven recruitment failure of sea urchins is driving kelp recovery in this region.

Studies throughout the global range of kelp forests show that top‐down forces may have a strong and controlling impact on sea urchins (Boudreau & Worm, 2012; Clemente, Hernández, Montaño‐Moctezuma, Russeli, & Ebert, 2013; Estes et al., 2004; Gudimov et al., 2003; Ling, Johnson, Frusher, & Ridgeway, 2009; Ling, Johnson, Ridgway, Hobday, & Haddon, 2009; Steneck et al., 2013, 2004). The occurrence and abundance of crabs and other mesopredators that consume sea urchins depend, in part, on fishing activities and concurrent changes in the abundance of predators at higher levels in the food web. Where larger predatory fish species (such as the Atlantic cod, *Gadus morhua*) have been overfished, the abundance of mesopredators (e.g., decapods) may increase to levels that trigger regime shifts further down the food chain (e.g., between sea urchins barrens and kelp forests). In the northwest Atlantic, Steneck et al. (2004), and Steneck et al. (2013) showed that *Cancer* spp. crabs became the new top predator of sea urchins after Atlantic cod populations were overfished and decimated. In the Pacific, Livingston (1989) found that an increase in the Pacific cod (*Gadus macrocephalus*) stock led to reductions of snow crab (*Chionoecetes opilio* and *C. bairdi*) stocks, although the effect on the red king crab stock was less clear. This increase in abundance of predators at low trophic levels due to reduced abundance of predators at higher trophic levels is termed mesopredator release (Prugh et al., 2009) and is a type of trophic cascade effect (Baden, Emanuelsson, Pihl, Svensson, & Aberg, 2012; Moksnes, Gullström, Tryman, & Baden, 2008).

The importance of predators on sea urchin survival and recruitment is unclear for Norwegian waters (Sivertsen, 2006). However, Fagerli et al. (2014) showed high predation rate by *C. pagurus* and *C. maenas* crabs on newly settled sea urchin recruits in mid‐Norway. Hence, warming water temperature and increased abundance of expanding crabs may either be driving recovery of kelp in sea urchin barrens or operating as a reinforcing feedback mechanism, maintaining the kelp forest state in mid‐Norway. In the northern part of the overgrazed area of Norway (70°N), kelp recovery has been hypothesized to be triggered by the extensive increase in the abundance and distribution of the red king crab (*Paralithodes camtschaticus*; Falk‐Petersen, Renaud, & Anisimova, 2011; Sundet & Berenboim, 2008). The red king crab was introduced from the Pacific to Atlantic Russian waters during the 1960s and has later spread westward into northern Norway. The species feeds on *S. droebachiensis* (Jørgensen & Primicerio, 2007; Pavlova, 2009) and occurs at high densities in areas where local fishers have reported a decline in sea urchins (Gudimov et al., 2003). Atlantic cod prey upon both red king crab and edible crab, but their overall impact on the abundance of crabs and sea urchins is unknown (Dvoretsky & Dvoretsky, 2009; Holt, 1890; Link & Garrison, 2002; Norderhaug et al., 2005; Steneck et al., 2013).

**Figure 2:**
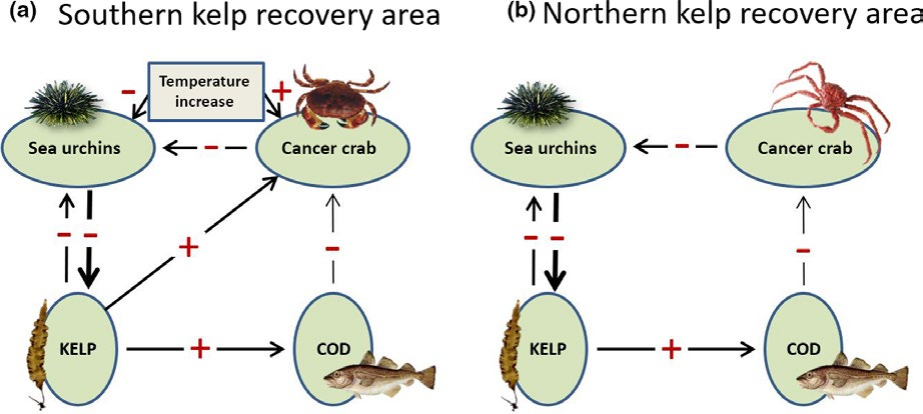
A conceptual model of the main interactions between key components in the kelp/sea urchin ecosystem (cf Table 1 for a detailed description of each interaction) in the (a) southern and (b) northern part of the kelp recovery area. Positive effects are marked by “+” and negative effects by “−”. The outlined interactions are based on previous studies and existing literature. The degree of support for each interaction (cf Table 1) is indicated by arrow thicknesses from thick (strong) to thin (weak)

The overall aim of this study was to explore possible links and interactions involved in the observed changes of the distribution of kelp forests and sea urchin barrens along the northeastern Atlantic coast of Norway and to relate these changes to possible drivers (ocean warming, changes in predator abundance). We constructed a conceptual model of the key species (kelp, sea urchins, crabs, and cod) and their possible interactions through postulated key processes (e.g., recruitment, predation, and fishing) and suggestions on how these species and interactions are modified by the proposed drivers (Figure 2). Through analysis of data from extensive field sampling and fisheries statistics, the relative abundance of the key species across spatial and temporal scales along the Norwegian coast is examined. Also, differences in kelp recovery and sea urchin recruitment between regions and how they possibly could be related to different climate and the availability of predator refuge habitats are explored. The conceptual model and the observed patterns are used to generate hypotheses on how multitrophic interactions and ocean warming may drive kelp recovery.

## 2 METHODS AND RESULTS

### 2.1 Statistical analyses

All statistical analyses were performed in R (version 3.5.0, R Core Team, 2018). For analyses testing linear relationships (correlations between cod and crab landings), the lm function in the *stats* library was used. Spatial patterns of kelp and sea urchin and sea urchin sizes, as well as sea urchin densities, were tested using generalized additive models (GAMs), which allow for nonlinear relationships where a nonparametric function is estimated for each predictor, to achieve the best prediction of the dependent variables. This was done using the library *mgcv* (Wood, 2017). For binomial responses (kelp and sea urchin probability), logit link functions were used. For count responses (sea urchin density), a Poisson distribution was assumed with a log link function. The remaining analyses assumed a normal (Gaussian) distribution; thus, an identity link function was used. When different candidate models were tested, the Akaike's Information Criterion corrected for small samples (AICc, Burnham, Anderson, & Huyvaert, 2010) was used to select the best model. To relate crab abundance to the occurrence of urchin barrens, we tested the proportion of barren vs. kelp recovery sites within areas with and without crab landings, using Pearson's chi‐squared tests, applying the prop.test() function in the *stats* library.

### 2.2 Kelp forest and sea urchin distribution patterns

**Figure 3:**
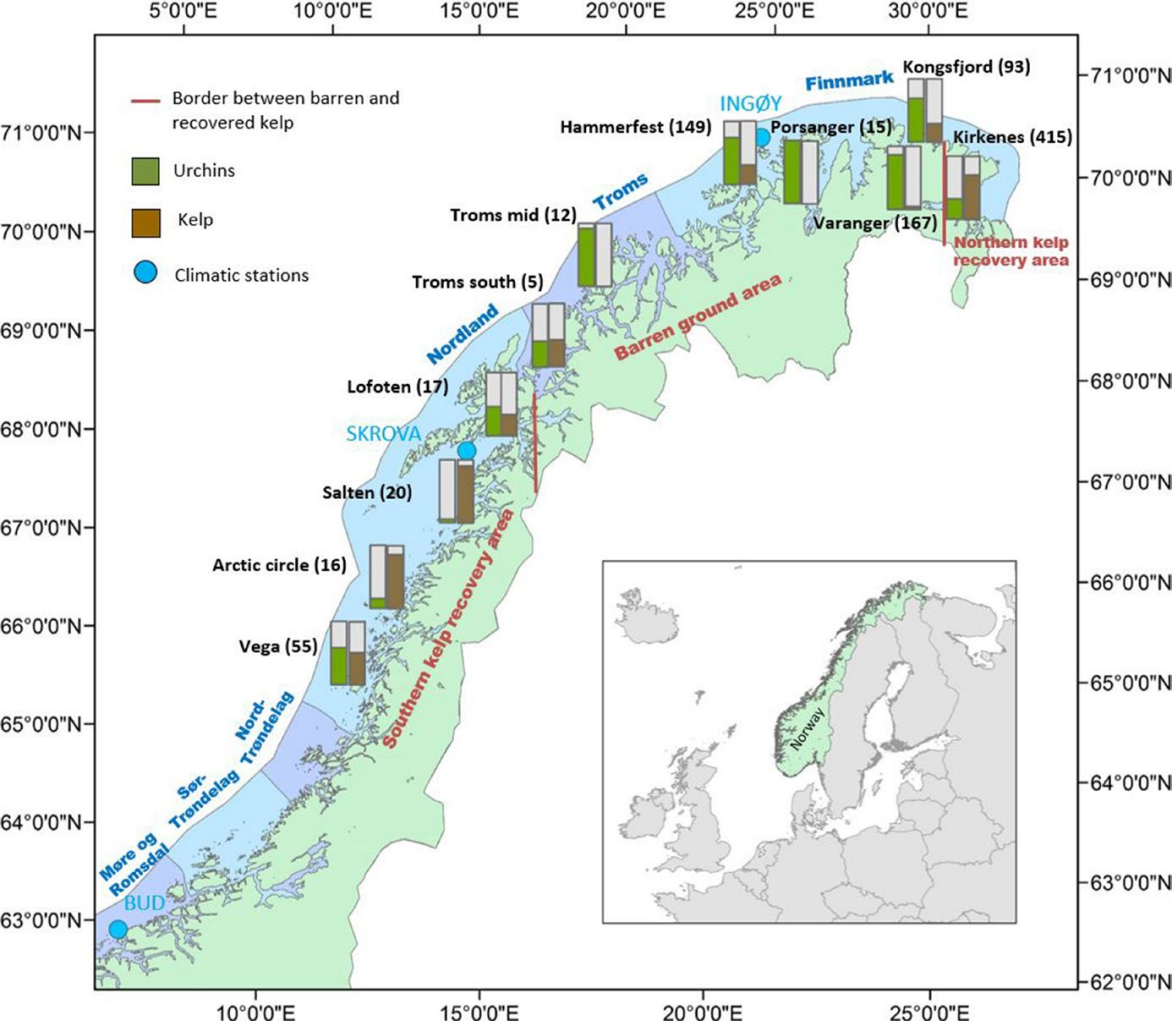
Map of northern Norway showing the distribution of the 11 sampling areas from Vega (~65.5^o^N, 12.5^o^E) to the Russian border in the northeast (and also north to ~71^o^N, 27^o^E) with relative abundance of sea urchins (*Strongylocentrotus droebachiensis*, green columns) and kelps (*Laminaria hyperborea* and *Saccharina latissima*, brown columns) based on a total of 1,249 stations. Presence of kelp and sea urchins is not mutually exclusive, and the total percentage might therefore exceed 100% in some areas. The number of stations in each sampling area is shown in brackets. The three climatic stations (Bud, Skrova, and Ingøy) are shown as light blue dots. Counties are shown as blue and violet sections along the coast. The borders between the barren ground area and the northern and southern kelp recovery area are indicated by dark red lines

A key pattern in the conceptual model of the large‐scale kelp recovery along the Norwegian coast (Figure 2) is that kelp recovery is occurring along the mid‐ and northern coasts of Norway. We hypothesize that this is due to reduced grazing by lowered densities of sea urchins, allowing kelp to recolonize the barrens (Figure 2; Sea urchin ↔kelp). To explore the distribution patterns of kelp forest and sea urchins, we used a spatially comprehensive dataset of kelp (*L. hyperborea *and *S. latissima*) and green sea urchin (*S. droebachiensis*) recordings from surveys performed between 2008 and 2012, from more than 1,500 km of the mid‐ and northern Norwegian coast (65–71°N, Figure 3). This part of the coast is morphologically complex with many islands and fjords. Kelp and sea urchin recordings were conducted at 11 sampling areas with 15–376 stations per area (average = 114, *SD* = 104), depending on boat time available, weather conditions, and presence of rocky habitat, totaling 1,249 stations, each with one replicate point observation (Figure 3 and Appendix A). The depth range was between 0 and 28 m (average = 6.4, *SD* = 4.8). The survey covered the three counties, Nordland, Troms, and Finnmark, from the 2007 southern limit of kelp recovery around Vega in the Norwegian Sea (Norderhaug & Christie, 2009) and northeast to the Russian border in the Barents Sea (Figure 3). All stations were situated on stable hard substrate, either bedrock or cobblestone/boulder (Wentworth, 1922, further called “cobblestone”), in areas that were previously grazed barren grounds with high densities of (visible) adult sea urchins (~20–50 individuals per m^2^) (Sivertsen, 1997; Skadsheim et al., 1995). The stations were in sheltered and moderately wave‐exposed areas (exposure value swm < 500,000, Bekkby, Rinde, Erikstad, & Bakkestuen, 2009, Gundersen et al., 2011). More wave‐exposed areas are generally not subject to sea urchin grazing (Rinde et al., 2014). The three southernmost sampling areas, Vega, Arctic Circle, and Salten, represent “the southern recovery zone,” identified by Rinde et al. (2014). The “northern recovery zone” was defined by the bounds of Kirkenes area, which was the only location with kelp recovery this far north in the present study. The seven sampling areas in between the two recovery zones (Lofoten, Troms south, Troms mid, Hammerfest, Porsanger, Kongsfjord, and Varanger) represent “the barren zone.” To explore the relationship between kelp and sea urchins, we calculated Pearson's correlation coefficient between the two variables (available as presence and absence data for each study site), for both the whole data set, and for each of the three zones (the two recovery areas and the barren zone). The surveys were performed between 2008 and 2012. Note that this spatially extensive dataset was compiled from many different surveys and projects, and different areas were covered each year, so the data are not balanced in space and time (see time schedule for field recordings in Appendix A).

Kelp and sea urchin abundance data were recorded from small boats using geographic positioning system (GPS) and underwater cameras equipped with depth sensors, giving us a view of the seabed with the extent of 1–2 m^2^. With few exceptions, the camera view either revealed pure dominance of kelp (i.e., kelp present, sea urchin absent) or pure dominance of sea urchins (i.e., sea urchin present, kelp absent). Only at a few stations, both species were present, possibly indicating a transitional state (i.e., hysteresis, Ling et al., 2015, Figure 1).

To create a single variable that captured the relative position of each station along the coastline (i.e., instead of using the two highly correlated variables latitude and longitude), we calculated the position at the coast as the linear distance from each station to a reference point around Troms mid (68.7°N, 20.4°E), with negative distances to sites south of this reference point and positive distances to sites east of it. We identified this reference point using a piecewise regression analysis using the *segmented* R library (Muggeo, 2008), correlating the station's latitude against longitude, which fits the coastline very well (single breakpoint = 20.44, *R* ^2^_adj_ = 0.98). This variable was then used to capture the spatial variation in the analyses of kelp and sea urchin presences, as well as sea urchin size distribution, along the coast.

Spatial patterns of kelp and sea urchin presences along the Norwegian coast were explored using mixed GAMs. Two binomial models related the presence of kelp and sea urchins to the position at the coast. Two candidate models were tested in each case, with and without year as a categorical covariable. A random factor was included to account for nonindependent observations within each sampling area.

### 2.3 The spatial pattern of kelp recovery

Both sea urchins (*S. droebachiensis*) and kelp (*L. hyperborea* and *S. latissima*) were found in all 11 sampling areas along the coast, although kelp was at very low occurrences in Troms mid and Porsanger (Figure 3, Appendix A). This implies an increased kelp recovery since 2007 (reported by Norderhaug & Christie, 2009), but persistence of the barren zone in Troms. In the southern recovery zone, kelp was observed at 51%, 86%, and 90% of the stations at Vega, Arctic Circle, and Salten, respectively (Appendix A). In the northern recovery zone (i.e., Kirkenes), kelp forests were observed at 71% and 68% of the stations visited in 2011 and 2012, respectively. The remaining sampling areas, representing more than 1,200 km of the coast, were still dominated by sea urchin barrens, even though *L. hyperborea* occurred at some of the most wave‐exposed shallow locations. Pearson's correlation coefficient between kelp and sea urchins for the whole study area, and for the southern, barren, and northern zone, respectively, was as follows: −0.82, −0.79, −0.85, and −0.72, indicating a negative correlation between the two in all areas.

**Figure 4:**
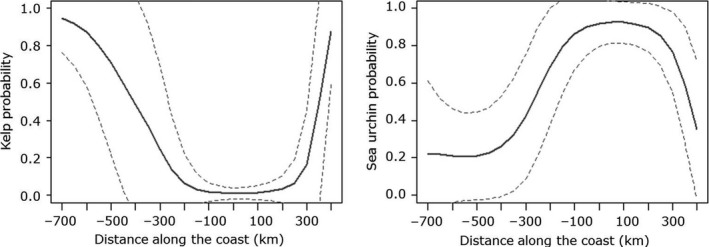
Predicted curves from the GAM models showing the opposite probability of occurrence of kelp (left) and sea urchins along the coast at cobble and bedrock bottoms. Sea urchin and kelp presence was mutually exclusive on all sampled stations. See Appendix A for how the distance along the coast relates to latitude and sampling areas

The GAM showed that the probability of finding kelp strongly related to the position along the coast (*F* = 5.66, *p* = 0.0002). The probability dropped from approximately one in the southern kelp recovery area to close to zero between −200 (Troms south) and 200 (Porsanger; Figure 4). From Porsanger toward the northern recovery area (Kirkenes), the probability of kelp increased sharply. The model with year included (*R* ^2^ = 0.25) was equally good as the one without (*R* ^2^ = 0.13, ΔAIC = 0.2). The opposite pattern was found for the sea urchins (Figure 4), but here, the model without year (*R* ^2^ = 0.18) was selected in favor of the one with year included (*R* ^2^ = 0.18), based on AIC values (ΔAIC = 10.7).

### 2.4 Sea urchins use of predator refuge habitats in the recovery zones

During initial surveys in the kelp recovery zones, we noticed that kelp recovery was limited to bedrock and that nearby cobble substrate remained overgrazed by sea urchins. This pattern could be a result of kelp preferentially settling on bedrock and not on cobble bottoms. However, Scheibling and Hamm (1991) found in a caging experiment that cobblestone habitats create spatial refugia for urchins that decrease predation by crabs. Based on this, we explored the sea urchins’ use of bedrock and cobblestone, which provide different levels of predator refuge for sea urchins and thus may indicate the influence of predators (Clemente et al., 2013; Falk‐Petersen et al., 2011). This was done at 185 stations within the kelp recovery zones in the south (i.e., Vega in 2012 and the Arctic Circle in 2011) and in the north (i.e., Kirkenes in 2012; Appendix A). All three areas have high crab landings (cf. the analysis of temporal and spatial patterns of changes in predator abundances below).

**Figure 5:**
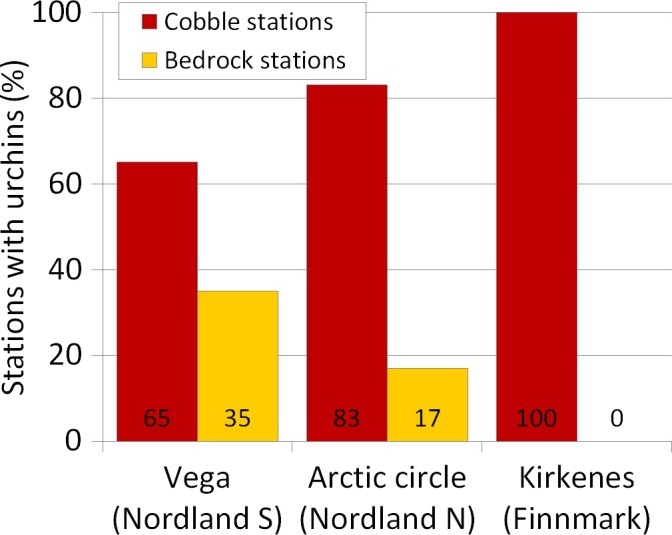
Percentage of stations with presence of sea urchins (*Strongylocentrotus droebachiensis*) within sampling areas in the southern (Vega, *n* = 41, and Arctic Circle, *n* = 16) and northern (Kirkenes, *n* = 33) recovery zones, on the two substrate types, bedrock and cobblestone bottoms, the latter serve as a predator refuge

Sea urchins were found on all cobblestone stations in each of the three areas (Figure 5). The frequencies of sea urchins on stations with open bedrock were 25% at Vega, 9% at the Arctic Circle, and 0% at Kirkenes. In Kirkenes, recovered kelp forests were found on 100% of the bedrock stations whereas 100% of nearby cobblestone stations were dominated by sea urchins in high densities (Figure 5).

Differences in occurrence of kelp and sea urchins between the sampling areas and between substrate types were tried tested in a binomial generalized linear model (GLM), but since there was no variation in the response in cobble substrate (sea urchin presence and kelp absence in all stations), the results are shown without any statistics.

### 2.5 Patterns of sea urchin recruitment success

Despite differences in temperature and nutrient supply, Fagerli et al., 2015 found that growth parameters for sea urchin populations from sea urchin barrens and kelp forests in the southern kelp recovery zone (Vega, 54°N) and the northern barren zone (Hammerfest, 70°N) were similar. Based on this premise, we investigated the sea urchin population structure using density and test size frequency as proxies for recruitment success and survival at 55 stations within the kelp recovery and barren zones (see station list in Appendix B). Occurrence of small juveniles in cryptic habitats indicates recruitment, and the occurrence of adults indicates survival from juvenile to adult stage.

All stations were at shallow depths (<10 m) and were defined as one of four different habitat types: bedrock (*n* = 24), cobblestone (*n* = 15), maerl (*n* = 10), and kelp holdfasts (*n* = 6; see Appendix B), where sea urchins are known to recruit (NIVA, unpublished data). By SCUBA diving, sea urchin density was estimated at each station by counting the number of individuals within 10 replicate frames of 50 × 50 cm (for bedrock or cobblestone bottoms), 4 replicate 20 × 20 frames (for maerl), or within four haphazardly collected kelp holdfasts (in areas with kelp forests). Sea urchins on bedrock and cobblestone were counted in situ*. *Sea urchins in maerl and holdfasts were collected while still within the substrate, then picked loose, counted, and size measured on land. For size frequency analysis of sea urchins at the bedrock and cobblestone stations, the first 200 individuals found within the frames were collected and the individual test diameter was measured with calipers on land. Sea urchins recruiting in kelp holdfasts were not converted to densities, as it is difficult to get exact data of area for this substrate.

Sea urchin size variation was also analyzed using mixed GAM at the level of individual sea urchins (*n* = 5,505), using station ID as a random factor to correct for possible nonindependence between individuals sampled from the same station. Six candidate models included different combinations of position at the coast, substrate, and year, and the interaction between the two latter. As holdfast and maerl did not provide sufficient data at several intervals along the coast (Appendix B) and thus resulted in huge confidence intervals, these two substrate types were excluded from the analyses of sea urchin size along the coast, and only cobblestone and bedrock were used.

Sea urchin density (number of individuals per m^2^) was tested for potential regional differences and substrate effects. Substrate types and zones (southern and northern recovery zone and barren zone) and their interaction were included in the model. Holdfast was excluded in this analysis, since density was not measured for this substrate type. Two candidate models were tested: one with additive effects, and one including an interaction between substrate and zone.

### 2.6 Spatial patterns of recruitment success

**Figure 6:**
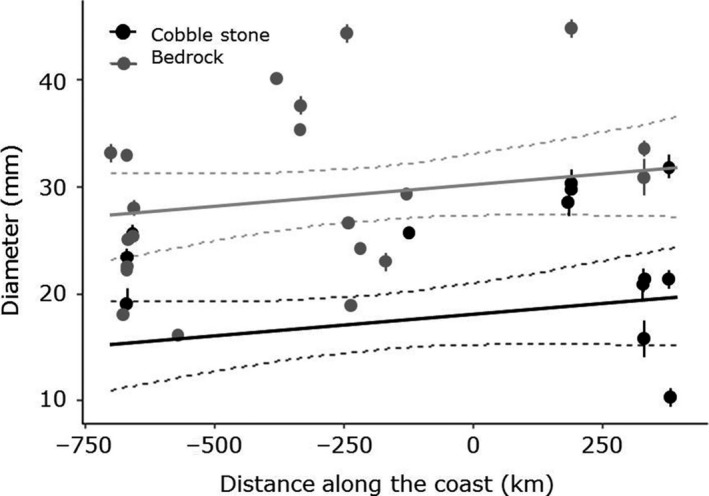
Average size (diameter ±2*SE*) of sea urchins (*Strongylocentrotus droebachiensis*) found on cobblestones (black) and bedrock (gray) along the coast. Neither maerl nor holdfast provided sufficient data to be included in the analyses. The dots show averages (±*SE*) at each sampling station. See Appendix A for how the distance along the coast relates to latitude and sampling areas

Small sea urchins were present in maerl and kelp holdfast within all the 55 studied stations (Appendix B), indicating that successful recruitment of sea urchins occurred across the 1,500 km study area. On average, small sea urchins were found within kelp holdfasts (8.0 mm ± 0.60) and in maerl beds (8.0 mm ± 0.69), while larger specimens were among cobblestones (25.0 mm ± 0.61) and on bedrock (29.0 mm ± 0.37). Sea urchin size (i.e., test diameter) was highly variable across the study area and not well explained by our explanatory variables (position at the coast, substrate, sampling year) in our selected mixed GAM (*R* ^2^ = 0.033, Figure 6). However, sea urchin size was significantly higher on bedrock compared to the cobblestone substrate (*p* < 0.001), but not significantly influenced by position at the coast (*p* = 0.069, Figure 6) or sampling year (*p* = 0.399).

**Figure 7:**
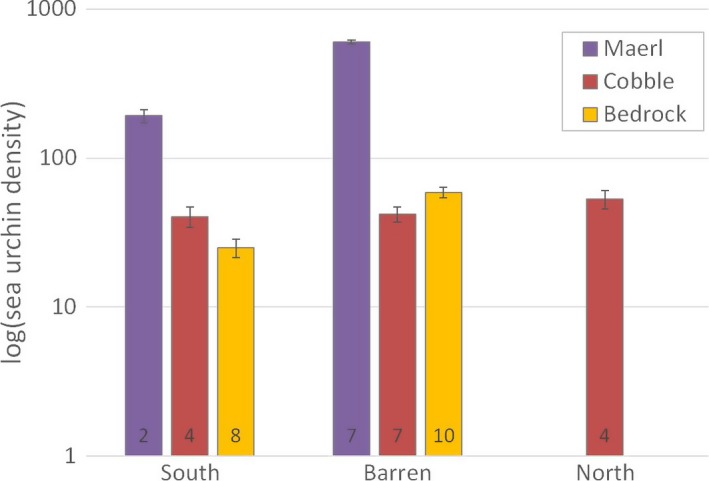
Average densities (predicted abundances per m^2 ^±2*SE* from GAM) of sea urchins (*Strongylocentrotus droebachiensis*) on three different substrate types (maerl beds, cobblestones, and bedrock) within the southern and northern recovery zones, and the barren zone (see map in Figure 3). The *y*‐axis is log‐transformed for illustrative purposes due to high sea urchin densities on maerl beds, particularly in the barren zone. The number of stations is shown at the column base

Densities were generally much higher in maerl beds than on cobblestones or bedrock, particularly at barren grounds in Troms and Finnmark (*χ* ^2^ = 7,304, *p* < 0.0001 for interaction between substrate and area, Figure 7) where the density of small (juvenile) sea urchins often exceeded several hundred per square meter (Appendix B). Average densities on maerl beds were 192 (*SD *= 70, *n* = 2) in the southern recovery zone and 599 (*SD *= 366, *n* = 7) in the barren ground zone. Average densities for cobblestones were 41 (*SD *= 46, *n* = 4) for the southern recovery zone, 42 (*SD *= 28, *n* = 7) for the barren zone, and 53 (*SD *= 41, *n* = 4) in the northern recovery zone. Average densities for bedrock were 25 (*SD *= 12, *n* = 8) in the southern recovery zone and 59 (*SD *= 41, *n* = 10) in the barrens zone. Densities were not measured on maerl and bedrock in the northern recovery zone.

### 2.7 Temporal and spatial patterns of changes in predator abundances

A hypothesized driver of sea urchin declines is the changing abundances of sea urchin predators (Figure 2; Crab → sea urchin; Cod → crab). Although a predator–prey interaction between crab and sea urchins (and between cod and crab) has been documented within the study area (Enoksen & Reiss, 2017; Fagerli et al., 2014), it remains unclear whether crab predators exert top‐down control on sea urchin populations. We explored spatial and temporal patterns of changes in abundance of the edible crab (*C. pagurus*) and red king crab (*P. camtschaticus*) based on landings from the period 1990–2011 (made available by the Norwegian Directorate of Fisheries), for the fishery zones in our study area. Data on landings are influenced by fishery effort and quotas and do not represent exact quantitative stock sizes or catch per unit effort. Nevertheless, these data are the best available indication of the size of the crab populations within the study area, as earlier reported by Woll et al. (2006) for *C. pagurus* and Sundet and Berenboim (2008) for *P. camtschaticus*. Changes in the abundance of these predators may be an indicator for changes of the level of predation pressure on sea urchins in our study area. To explore the link between abundance of crab and the state of the system, we performed Pearson's chi‐squared test of the proportion of sites with barren vs. kelp recovery state, within the identified regions with and without crab landings. Thirty years of data on the abundance of the Norwegian coastal cod (*G. morhua*) stock north of 62°N (Berg, 2012; ICES, 2004) were also available. Note that coastal cod and the NE Atlantic Arctic/pelagic cod are the same species but are from genetically distinct populations (Westgaard & Fevolden, 2007).

**Figure 8:**
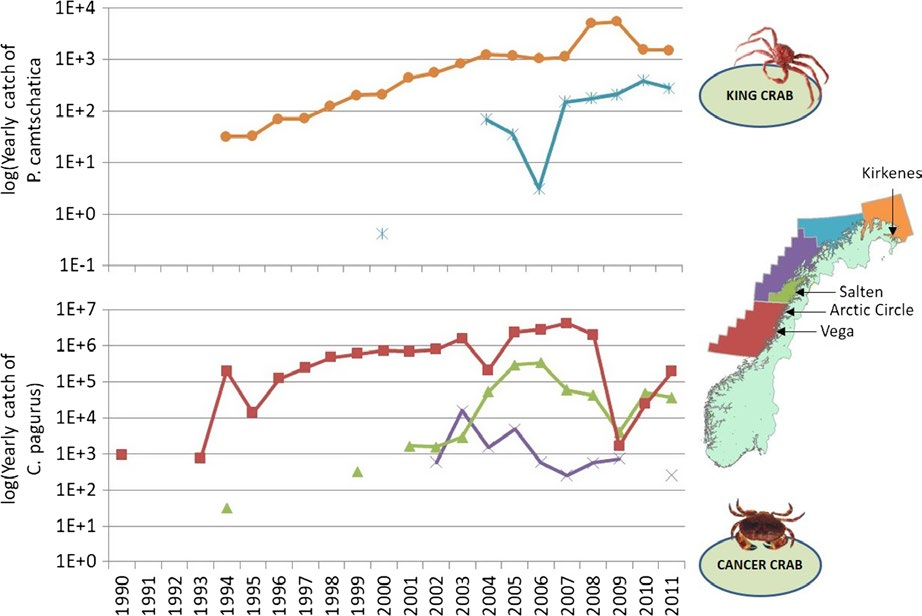
Landings of red king crabs (*Paralithodes camtschaticus, *upper panel) and edible crab (*Cancer pagurus*, lower panel) within different fisheries zones from the 1990s to 2011. The color codes of the curves match the fishery zones. Data are from the Norwegian Directorate of Fisheries. *Y*‐axis is log‐transformed to show temporal variation also at low catch levels

The landing statistics indicate that edible crab (*C. pagurus*) abundances have increased since the early 1990s (Figure 8). Landings were highest in the southern fishing zone (red zone in Figure 8), which corresponds to the Arctic Circle and Vega areas in the southern kelp recovery zone. Landing data also suggest that edible crabs have expanded northward, appearing for the first time in the two northern adjacent fishing zones in 1994 (i.e., in the innermost green zone corresponding to the sampling area Salten in the southern recovery zone), and in 2002 (the offshore, adjacent purple zone, corresponding to the barren zone, as illustrated in Figure 8). There was a clear reduction in the landings of edible crab in 2009 (Figure 8) probably due to a cadmium contamination resulting in reduced fishing activity (Norwegian Fishery authorities). There is no landing statistics for edible crab for the two northernmost zones, most likely due to scarce occurrences of the species. Pearson's chi‐squared test revealed a high proportion of barren sites (95% confidence interval 0.74–1.0) in the barren zone region with low crab landings, and a low fraction (95% confidence intervals 0–0.32 and 0–0.35) in the recovery zone south and north, respectively, with high crab landings.

The catch of red king crabs (*P. camtschaticus*) has increased in Norwegian waters through the same period, and as much as 5,000 tons per year has been landed in the fishery zone closest to Russia (the orange zone in Figure 8), which corresponds to the Kirkenes area in the northern kelp recovery zone. More recent landings in the region further west indicate a gradual expansion of the species’ distribution westward into the barren zone (the blue zone in Figure 8).

**Figure 9:**
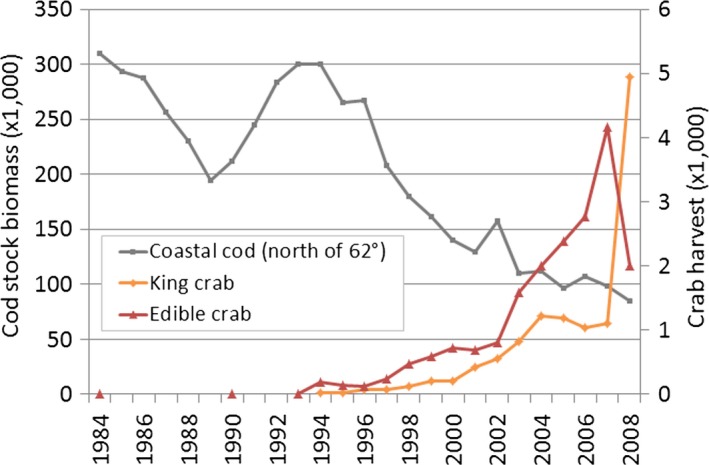
Temporal trends of coastal cod (*Gadus morhua*) stocks north of 62^o^N from 1984 to 2008 (Berg, 2012), and of king crab (*Paralithodes camtschaticus*) landings in eastern Finnmark, and edible crab (*Cancer pagurus*) landings in south Nordland. The color codes of the crab curves match the fishery zones in Figure 8 and coastal cod north of 62^o^N includes all fishery zones shown in Figure 8. Data are from the Norwegian Directorate of Fisheries

The Norwegian coastal cod (*G. morhua*) stock north of 62°N has decreased by 2/3 from 1993 to 2008 (Figure 9, data from Berg, 2012), a decline that coincides with the increase in both edible crab (*p* < 0.0001, *R* ^2^ = 0.75) and king crab (*p* = 0.0015, *R* ^2^ = 0.55) landings. The correlations between cod and edible and red king crab were calculated after log‐transforming crab data to achieve normally distributed residuals.

### 2.8 Temporal and spatial trends of changes in sea temperature

**Figure 10:**
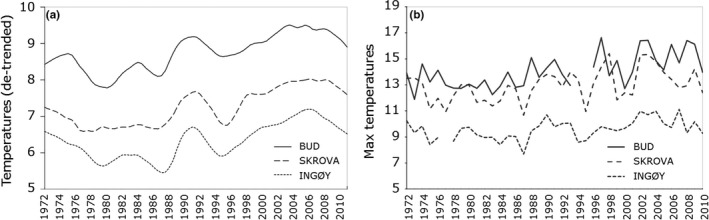
Detrended seasonal averages (a) and yearly maximum (b) sea surface temperatures (SST, measured at 1 m depth) from climatic stations at Bud (63°N), Skrova (68°N), and Ingøy (71°N; see map in Figure 3)

Temperature has been implicated in both recruitment failure of sea urchins and the expansion of cancer crabs (Figure 2; Temperature → crab, Temperature → sea urchin). To evaluate whether the documented range expansion of the crab *C. pagurus *(cf. Woll et al., 2006, Brattegard, 2011) and the collapse of *S. droebachiensis* populations are likely to be driven by temperature change (cf. Stephens, 1972), we explored time trends in the ocean climate along the south–north gradient within the study area over the last 38 years. We obtained temperature data from the three meteorological stations: Bud (63°N), Skrova (68°N), and Ingøy (71°N) (see Figure 3 for the positions of the hydrographical stations and Figure 10 for mean and maximum temperature). Monthly sea temperatures measured at 1 m depth from 1972 to 2010 were available from Albretsen, Aure, Sætre, and Danielssen (2011) for these three stations. Potential time trends in the climatic data were tested by the Mann‐Kendall trend tests for the Bud, Skrova, and Ingøy time series using the R library *Kendall* (McLeod, 2011).

Sea surface temperatures are generally lower at higher latitudes. There has been a significant increase in the temperature from 1972 to 2010 at all three climate stations (tau > 0.154, *p* < 0.0017, Figure 10). From 2000s, the Lofoten area (i.e., Skrova) has experienced temperatures like those observed in mid‐Norway (i.e., Bud) 20–30 years earlier (Figure 10a). During this time, the maximum sea surface temperature exceeded 15°C at the two stations in southern and middle part of the study coastline but remained below 11°C in the northernmost region (Figure 10b).

## 3 DISCUSSION

In the last half‐century, threats against kelp forests have increased globally, leading to worldwide declines of ~2% per year (Wernberg et al., 2018), with particularly extensive losses in some regions (Filbee‐Dexter & Wernberg, 2018; Krumhansl et al., 2016; Ling et al., 2015; Moy & Christie, 2012; Norderhaug & Christie, 2009; Raybaud et al., 2013; Smale, Burrows, Moore, O'Connor, & Hawkins, 2013; Steneck & Johnson, 2013; Wernberg et al., 2011). This has been documented to be related to increased sea temperatures, either directly due to heat waves (Wernberg et al., 2016) or through interaction between ocean warming and changes in predation pressure (Johnson et al., 2011; Ling, Johnson, Ridgway et al., 2009; Steneck et al., 2004; Watson & Estes, 2011). Contrary to this global trend, kelp *recovery* has taken place in Norway, in the southern and northern parts of previously grazed kelp areas, resulting in an increased kelp recovery compared to the situation reported for 2007 (Norderhaug & Christie, 2009). There is strong support from prior research that the recovery in the southern part of the overgrazed area is related to indirect and direct effects of ocean warming on sea urchins (Fagerli et al., 2013, 2014; Rinde et al., 2014). However, in this study we also document kelp recovery in the northernmost, coldest part of the overgrazed area, where the shift is not facilitated by temperature. Based on coinciding patterns of the spatial and temporal distribution of the key species (kelp, sea urchins, crabs, cod) and suggested processes involved (kelp recovery, sea urchin recruitment, sea urchin predation, sea urchins use of refuges), we hypothesize how multitrophic interactions and ocean warming can generate the observed patterns of large‐scale kelp recovery. The conceptual model summarizes the suggested interactions and forms an outline for further research and understanding of the ecosystem drivers. The strength of support to the postulated relationships behind the arrows in the model (Figure 2), based on other studies, is summarized in Table 1.

**Table 1:** Postulated links and interactions between key species in the conceptual model of the studied kelp/sea urchin ecosystem, including the hypothesized impact of ocean warming (i.e., just the southern recovery area) and crabs on sea urchins in the two recovery areas (a southern and a northern area). Observed patterns from this study (if any) and the type of observation/statistic are described. The degree of support for the hypothesized links from other kelp–urchin studies are classified into strong, medium, or weak, and as causal or correlative. The references of the other studies are given

Interaction/Description	Degree of support other studies	Observed patterns in this study	References
Sea urchin ↔ kelp			
1. Green sea urchins in high densities are grazing kelp and maintaining barren grounds	Strong causal	Negative correlation between kelp and sea urchin occurrence	Norderhaug and Christie (2009), Propp (1977), Rinde et al. (2014), Skadsheim et al. (1995), and Sivertsen (1997, 2006)
2. Kelp forests house sea urchin predators that regulate sea urchin abundance	Medium correlative	Sea urchins rarely observed inside kelp forests	Norderhaug and Christie (2009), Skadsheim et al. (1995), and Steneck et al. (2013)
Kelp → crab			
3. Kelp forests are habitat for *Cancer* crabs	Strong causal		Christie, Fredriksen, and Rinde (1998), Fagerli et al. (2014), Steneck et al. (2013), and Woll et al. (2006)
Crab → sea urchin			
4. *Cancer* crabs feed on sea urchins	Strong causal	Observations in field	Fagerli et al. (2014), and Steneck et al. (2013, 2004)
5. *Cancer* crabs reduce sea urchin populations	Correlative	An inverse pattern of abundance in time and space. Low proportion of barrens in areas with crab landings, and high proportions in areas without.	Steneck et al. (2013, 2004)
6. Red king crabs feed on sea urchins	Strong causal	Observations in the field	Gudimov et al. (2003), Jørgensen and Primicerio (2007), Oug and Sundet (2008), and Pavlova (2009)
7. Red king crabs reduce sea urchin populations	Correlative	An inverse pattern of abundance in time and space of crab landings and sea urchin density. Sea urchins on predator refuge habitats	Gudimov et al. (2003) and Oug and Sundet (2008)
Kelp → cod			
8. Kelp forests are a habitat for coastal cod, particularly juveniles	Strong causal	Own unpublished results	Keats et al. (1987) and Norderhaug et al. (2005)
Cod → crab			
9. Coastal cod feed on edible crabs	Strong causal	Observations in the field	Holt (1890), Link and Garrison (2002), Norderhaug et al. (2005), and Steneck et al. (2013)
10. Coastal cod population size influences edible crab populations	Weak correlative	An inverse pattern of abundance in time and space of cod and Cancer crab landings.	Steneck et al. (2013) for *Cancer* spp.
11. Coastal cod feed on king crabs	Strong causal		Dvoretsky and Dvoretsky (2009), Falk‐Petersen et al. (2011), and Livingston (1989)
12. Cod population size influences the size of king crab populations	Weak correlative	An inverse pattern of abundance in time and space of cod and king crab landings	Livingston (1989) for snow crabs
Temperature → sea urchin			
13. Temperature increase is negative for sea urchins	Strong causal	Temporal and spatial correlations in the mid‐Norway	Fagerli et al. (2013, 2014), Rinde et al. (2014), Stephens (1972), and Siikavuopio et al. (2006, 2012)
Temperature → crab			
14. Temperature increase is positive for the edible crab and results in northward movement of the crab	Medium correlative	Temporal and spatial correlations in the mid‐Norway)	Brattegard (2011), Lindley and Batten (2002), Lindley and Kirby (2010), and Woll et al. (2006)

We have documented kelp recovery in areas previously grazed by sea urchins in a recently warmed area (southern study area) and in a still cold area (northern study area). The high negative correlations between kelp and sea urchins (>−0.72) indicate a causal, negative relationship between the two response variables (see also Filbee‐Dexter & Scheibling, 2014, Ling et al., 2015). Hence, we hypothesize that ocean warming and expansion of sea urchin predators, such as crabs, explain the spatial pattern seen for kelp recovery. The analysis of crab landings indicates that the abundance of crabs has increased in the recovery areas. We hypothesize that this increase (in edible crab in the southern area) is made possible by both warmer water and the decrease in the abundance of cod (in accordance with the mesopredator release hypothesis). The analysis of sea urchin juvenile density patterns indicates that sea urchin recruitment rate is increasing toward the north, indicating a negative influence of warm water on sea urchin recruitment. However, sea urchins recruit successfully also in the southern study area, as manifested by the presence of barren patches with high sea urchin densities, including the presence of juveniles. To explain the observed mosaic pattern of kelp and barren grounds in both recovery areas (the warm south and the cold north), we hypothesize that the sea urchins can maintain these barrens due to presence of structurally complex substrates, like cobblestones, that provide refuge from predators. Lack of kelp recovery in the intermediate part of the study area (Troms/Varanger), combined with cold temperature and low crab densities in this area (as indicated by our analysis of fishery landing), suggests the following hypothesis: The cold water enhances sea urchin recruitment, and the sea urchins are not controlled by crab predation, leading to sea urchin densities above the threshold density needed to sustain barren conditions and preventing kelp recovery. Support for the described patterns is explained below.

The high crab abundance within the kelp recovery areas, as well as substantial observations of sea urchins using predator refuge habitats in these areas (as also found by Clemente et al., 2013 and Scheibling & Hamm, 1991), indicates that top‐down regulation of the sea urchins by edible crab and king crab predation may have occurred within the study area. Kelp recovery in the south and in the north correlates both in time and in space with the increased crab catches (Figure 8), which indicate a northward migration by the edible crab (and possibly also by the green crab, Fagerli et al., 2014) in the south, and a westward expansion of the invasive red king crab in the north. In the northernmost and coldest region with high king crab landings, high recruitment and high densities of sea urchins only occurred in predator refuge areas and kelp had recovered in many stations (Kirkenes area, close to the Russian border).

In contrast, the barren zone (region with high frequency of sea urchin‐dominated stations) had low levels of crab landings and high levels of sea urchin recruits on all substrates. These extensions of crab populations have previously been shown for the edible crab, *C. pagurus* (Brattegard, 2011; Woll et al., 2006) and the red king crab, *P. camtschaticus* (Falk‐Petersen et al., 2011; Sundet & Berenboim, 2008). Although crab landings depend on fishery effort, quotas, and market, and therefore do not always reflect the background population size, the expansion over time in density and in spatial distribution of both crab species is supported by anecdotal accounts from fishers and divers in these regions. Finally, stomach content analyses of red king crabs (Gudimov et al., 2003; Jørgensen & Primicerio, 2007; Oug & Sundet, 2008; Pavlova, 2009) and laboratory and field experiments on edible crabs within the southern recovery area (Fagerli et al., 2014) showed that both crab species feed on green sea urchins (*S. droebachiensis*). Thus, there is strong support to suggest that predation rates have reduced sea urchin abundances to a level that facilitates a reverse shift to kelp recovery in some areas (see also Clemente et al., 2013; Falk‐Petersen et al., 2011).

The increase in crab abundance has been attributed to both ocean warming and reduced pressure from top predators. The negative correlation between coastal cod stock abundance and the crab landings in the study area is in line with the hypothesis that mesopredator release of crabs due to the coastal cod fishery may have occurred in the NE Atlantic. Atlantic cod preys upon edible crabs (Holt, 1890; Norderhaug et al., 2005; Ungfors, 2008) and red king crabs (Dvoretsky & Dvoretsky, 2009; Falk‐Petersen et al., 2011; Livingston, 1989). The coastal cod was previously a dominant top predator in coastal areas of Norway, but the population north of 62^o^N has been reduced by about two‐thirds since the mid‐1990s (Berg, 2012; ICES, 2004). Similar top‐down mechanisms, in which overfishing and reduced abundance of exploited fish stocks contribute to ecosystem changes in coastal regions, were demonstrated in the NW Atlantic (Steneck et al., 2013), which has the same species or genus of kelp, sea urchin, crabs, and cod as the NE Atlantic (Jackson et al., 2001; Steneck et al., 2013). Effects of change in the top predator population size have been clearly linked in time and space by Livingston (1989) where the increasing Pacific cod (*G. macrocephalus*) stock led to reduced snow crab (*C. opilio* and *C. bairdi*) stocks. However, this link remains to be tested in Norway, and data on crab and cod catch per unit effort are required at local levels to robustly study this link.

Fagerli et al. (2013, 2014, 2015) and Rinde et al. (2014) found increasing ocean summer temperature to be an important factor contributing to reduced abundance of sea urchins. The influence of ocean warming on sea urchin abundance is in our model assumed to occur through a direct negative impact on growth and reproduction, and indirectly, through the northward movement of the edible crab (Table 1). The summer temperatures (Figure 10) north to Lofoten are close to the critical threshold temperature for the cold‐water sea urchin *S. droebachiensis *(Stephens, 1972) and may introduce physiological stress resulting in reduced growth and reproduction of this species (Siikavuopio et al., 2006, 2012). The low density of juvenile sea urchins in maerl beds in the south compared to the north is in line with the hypothesis of a negative influence of warm water on sea urchin recruitment (Fagerli et al., 2013).

In general, both kelp forests and barren grounds are considered stable states that require a certain amount of disturbance to be transformed to the other state (e.g., Ling et al., 2015; Scheffer et al., 2001). There are other unexplored feedbacks that may also be at play in this system. For example, kelp recovery will increase shelter and feeding grounds for juvenile cod (Keats, Steele, & South, 1987; Norderhaug et al., 2005), which may lead to increased predation on crabs (as demonstrated by the cod and crab relationship by Livingston, 1989), and thereby reduced predation pressure on sea urchins. This may enhance new blooms of sea urchins in the north if red king crabs are sufficiently reduced (by predators or heavy fisheries). Ocean warming might increase the resilience of the restored kelp forests in the southern area (Figure 2), due to its negative impact on sea urchins and positive impact on edible crabs; that is, if ocean warming does not have a negative impact on the performance of kelp. Both *L. hyperborea* and *S. latissima* will most likely be able to cope with the temperature increases projected for these cold northern parts of the NE Atlantic, even though kelp forests are predicted to be reduced due to global climate change affecting rates of carbon assimilation (Moy & Christie, 2012; Pessarrodona, Moore, Sayer, & Smale, 2018). The positive or negative interactions hypothesized in Figure 2 indicate how an increase or a decrease in one trophic level will affect the other trophic levels. However, before this conceptual model can serve as a tool for management of the system, the causative relationships must be established so that managers can target relevant monitoring parameters. With further data collection, the large persistent overgrazed area between the two kelp recovery areas represents a great potential for a kelp forest recovery zone that can serve as a future test area of the higher trophic‐level populations and their interactions.

While the causal relationships between species at the individual level (e.g., predation, facilitation) can be observed and tested (interactions 1, 3, 4, 6, 8, 9, 11, and 15 in Table 1), it is more uncertain how these relationships can be extrapolated to the population level. The data presented in this study provide only correlations between high crab landing data and a low frequency of sea urchin barrens (interactions 5 and 7 in Table 1). However, that such relationships exist are supported by findings in other studies and areas (see references in Table 1). Similarly, the relationship between the decline in the Norwegian coastal cod and the increase in crab populations (interaction 10 and 12 in Table 1) and the relationship between temperature increase and the northward expansion of *C. pagurus* (interaction 14 in Table 1) are based on correlations, as is most of the support from existing literature (Table 1). To confirm that multitrophic interactions are occurring at the population level, adjacent areas with different population sizes of the top predators must be found, as in the study of Hughes et al. (2013) and Baden et al. (2012).

A repeated question has been what caused the initial bloom of sea urchins along many temperate coasts (Elner & Vadas, 1990; Sivertsen, 2006). This study cannot provide answers to the causes of blooms of sea urchins many decades ago but may contribute by pointing at factors that are important for regime shifts and resilience of the kelp forest and sea urchin barrens states in this system. Complex interactions such as recruitment success, altered predation pressure, and environmental factors (mainly temperature) may have favored the sea urchins at the time of outbreak.

## CONFLICT OF INTEREST

None declared.

## AUTHOR CONTRIBUTIONS

All the authors have made substantial contributions to the content of the manuscript.

## Data Availability

Data supporting the results in the paper are uploaded and archived in Dryad.

## APPENDIX A

Percentage of stations with presence of kelp (*Laminaria hyperborea *and/or *Saccharina latissima*) and sea urchins (*Strongylocentrotus droebachiensis*) within each of the 11 sampling areas. All stations are in sheltered and moderately exposed hard bottom areas recorded with underwater camera in previously grazed barren grounds of mid‐ and northern Norway. Three of the areas (Hammerfest, Porsanger, and Kirkenes) were sampled two consecutive years. The two species are not mutually exclusive, and total percentage might therefore exceed 100%

**Table:**

Area	Year	Latitude	Dist. along coast	No. of stations	% of stations with kelp	% of stations with sea urchins
Vega	2012	65.7	−674	55	51	58
Arctic Circle	2011	66.5	−574	90	86	16
Salten	2011	67.4	−447	89	90	6
Lofoten	2011	68.3	−333	63	33	46
Troms south	2011	69.0	−241	71	42	41
Troms mid	2011	69.8	−131	24	0	92
Hammerfest	2008	70.8	115	123	39	64
Hammerfest	2009	70.7	115	83	18	88
Porsanger	2010	70.4	188	10	0	100
Porsanger	2011	70.4	188	5	0	100
Kongsfjord	2012	70.7	328	91	29	69
Varanger	2012	70.1	334	169	5	86
Kirkenes	2011	69.9	372	336	71	32
Kirkenes	2012	69.9	372	40	68	35

## APPENDIX B

Density (abundance per m^2^) and test size distribution of sea urchins, *Strongylocentrotus droebachiensis*, recorded quantitatively at different substrate types by SCUBA diving. Density estimates in kelp holdfasts were not measured, since it is difficult to get an exact estimate of area in this substrate

**Table:**

Area	Substrate type	Density (*n*/m^2^)	Mean size (mm)	Size range (mm)
*Southern recovery zone*				
Vega				
Torghatten	Bedrock	9	33	7–50
Torghatten	Maerl beds	241	6	2–12
Rørøy	Maerl beds	0	na	na
Søla	Bedrock	44	18	4–35
Sandøy N	Cobble stones	79	24	8–44
Sandøy N	Bedrock	21	33	11–44
Andholmen	Bedrock	20	23	13–46
Andøy	Bedrock	24	25	9–46
Skogsholmen	Bedrock	42	28	11–54
Skogsholmen	Bedrock	25	25	14–49
Skogsholmen	Cobble stones	81	26	10–47
Skogsholmen	Maerl beds	142	11	3–19
Tuvøy	Bedrock	15	22	10–44
Tuvøy	Cobble stones	2	19	9–37
Igerøy	Kelp holdfast	0	na	na
Arctic Circle				
Hestmann	Bedrock	26	16	4–31
*Barren zone*				
Lofoten				
Lyngvær	Bedrock	19	40	2–63
Lødingen	Bedrock	14	38	9–61
Tysfjord	Bedrock	36	35	21–48
Troms				
Meløyvær	Bedrock	8	44	9–60
Løksefjord	Bedrock	35	27	10–46
Musvær	Bedrock	64	29	9–46
Kvalsund	Cobble stones	23	26	13–48
Buvika	Bedrock	21	23	5–49
Buvika	Maerl beds	575	16	2–63
Leirpollen	Maerl beds	150	12	2–19
Leirpollen	Bedrock	82	20	9–31
Leirpollen	Bedrock	23	18	7–34
Hyseskjær	Maerl beds	716	6	1–14
Humpen	Maerl beds	725	5	1–15
Humpen	Bedrock	17	24	7–44
Lemmingsver	Kelp holdfast	na	8	3–14
Flua	Kelp holdfast	na	8	3–17
Senja, inner	Kelp holdfast	na	7	3–13
Senja, outer	Kelp holdfast	na	10	5–17
Porsanger				
Hamnholmen	Bedrock	56	45	17–63
Hamnholmen	Cobble stones	23	30	9–51
Veineset	Cobble stones	37	29	4–68
Hamnholmen	Cobble stones	45	30	3–69
Hamnholmen	Bedrock	154	45	25–58
Kongsfjord				
WP 298	Maerl beds	1,225	6	3–16
WP 325	maerl beds	175	9	2–32
WP 353 (Kua)	Cobble stones	21	21	2–64
WP 357	Bedrock	79	31	3–54
WP 357	Cobble stones	101	15	3–58
WP 356	Bedrock	58	34	16–49
WP 356	Cobble stones	45	21	7–50
WP 358	Maerl beds	629	5	2–28
*Northern recovery zone*				
Kirkenes				
WP 54	Cobble stones	20	32	8–62
Kjelmøy	Cobble stones	103	14	4–37
Kjelmøy	Cobble stones	18	28	4–60
Kjelmøy	Cobble stones	71	na	na
Kjelmøy	Bedrock	0	na	na
WP 89	Cobble stones	na	10	6–26
WP 89	Kelp holdfast	na	7	3–13
